# Special Issue “Mesenchymal Stromal and Immune Cells’ Involvement in Human Diseases and Their Treatment”

**DOI:** 10.3390/ijms27020850

**Published:** 2026-01-15

**Authors:** Mirjana Jerkic

**Affiliations:** The Keenan Research Centre for Biomedical Science of St. Michael’s Hospital, Unity Health Toronto, University of Toronto, Toronto, ON M5B 1W8, Canada; mirjana.jerkic@unityhealth.to

Mesenchymal Stromal Cells (MSCs), defined as multipotent, fibroblast-like, non-hematopoietic cells [1], have the ability to differentiate into various mature cell types, including adipocytes, chondrocytes, osteoblasts, and myoblasts. MSCs have been isolated for therapeutic purposes from many sources, of which bone marrow [2], umbilical cord [3], adipose [4], perivascular [5], and dental pulp [6] tissue are most frequently used. Different factors can influence MSC properties and their therapeutic efficacy. This Special Issue highlights the importance of cross-talk between MSCs, immune cells, and their environment in various disorders and disease treatments. 

MSC–immune cell interactions include, but are not restricted to, monocytes/macrophages [7,8], as well as dendritic, T, and B lymphocytes [9], with regulatory and memory T cell compartments playing a key role [4,10]. MSCs also have the potential to mitigate autoinflammation in the diseased microenvironment [11]. Recent studies, conducted in preclinical models and clinical trials, have demonstrated that investigating the mechanisms of MSC–immune cell–environment interactions has tremendous potential for optimizing MSC therapeutic effects [12,13,14,15,16,17].

Along these lines, Younesi and Hinz (Contribution 1) have focused on the myofibroblast fate of therapeutic MSCs and the fact that ex vivo amplification could reduce the desired MSC regenerative potential because of their conversion into fibrogenic myofibroblasts. This usually happens upon MSC contact with stiff cell culture plastic or recipient scar tissue. Mechanical stress, detected through mechanoperception mechanisms, triggers transcriptional programs such as YAP and TAZ (primarily controlled by the Hippo pathway) [18], as well as myocardin-related transcription factor (MRTF), whose activation can induce lasting epigenetic modifications at the DNA level. The authors discussed how modulating MSC culture could protect the cells from myofibroblast activation by preconditioning (priming) MSCs on soft culture surfaces. As an alternative to the cell culture priming, an alteration of MSC memory formation could enhance MSC beneficial effects. The authors have shown in their previous experiments that knocking down the myofibroblast keeper gene miR-21 could restore the regenerative capabilities of stiff-primed MSCs [19]. Other possibilities include manipulation of epigenetic DNA changes or epitranscriptomic RNA transformations [20]. All these manipulations could generate MSCs with enhanced regeneration potential and ensure their clinical therapeutic benefits.

On the other hand, aging MSCs could induce unfavorable changes in their environment. The processes behind MSC senescence remain unexplored, with the exception of phenotypic changes observed in aging MSCs [21,22]. However, how senescence-associated alterations in MSCs affect surrounding cells is poorly explored, with breakthrough advances made by the Ratushnyy Lab. Their previous work revealed that aging MSCs induce changes in the extracellular matrix (ECM) by altering endothelial cell activity [23]. Their work, published in this issue, evaluated senescence-associated changes in the matrisome of MSCs, including the negative impact of aging MSCs on ECM gene and protein composition, as well as the expression of secreted factors (Contribution 2). Aging MSCs induce downregulation of the most important structural and adhesion proteins, such as collagens, fibulins, and fibrilins, while the MSC secretome is shifted towards pro-inflammatory and pro-fibrotic proteins. Therefore, the modification of ECM proteins in the MSC microenvironment could slow down aging processes and improve tissue regeneration.

MSC interaction with the tumor environment is explored in the review paper by Gładyś et al. (Contribution 3). In particular, the authors summarized the prospects of improving the treatment of aggressive cancers, such as hepatocellular carcinoma (HCC). They explored the potential of using adipose-derived stem cells (ADSCs) in such treatments. The authors emphasized that the studies conducted so far have focused on the effects of ADSCs in cancer models, without exploring immunomodulation and ADSC interactions with the tumor ECM. Furthermore, there are no clinical studies evaluating the use of ADSCs in HCC or describing their role in other invasive cancers. Regardless of the possible pro- and anti-cancer effects of ADSCs, a better understanding of stem cell interactions with their environment will allow for the proper modification and use of ADSCs or their exosomes in cancer treatment. This could be a promising addition to standard therapies, due to the regenerative potential of ADSCs, their positive effect on ECM remodeling, and their direct effects on HCC cells.

Castillo-Galán et al. investigated potent antiviral properties of Umbilical Cord MSCs (UC-MSCs) triggered by activation of the mitochondrial antiviral signaling protein (MAVS), a crucial component of innate immunity [24]. The authors used Influenza A virus-derived 5′triphosphate-RNA (3p-hpRNA) to activate MAVS in UC-MSCs (Contribution 4). Subsequently, MAVS triggered the secretion of interferons (IFNs), IFN-stimulated genes, and pro-inflammatory cytokines. Critically, mitochondrial integrity and the capacity of UC-MSCs to transfer their mitochondria to recipient cells were preserved. This study highlights the importance of the immune response and mitochondrial transfer in MSC-based interventions against viral infections.

The potential of UC-MSCs to regulate B-cells, especially in autoimmune diseases, was explored by Yordanova et al. (Contribution 5). B lymphocytes actively participate in the etiology of autoimmune disorders [25] and represent an important target for the development of new treatment strategies for autoimmune disorders [26]. The authors (Contribution 5) assessed the immunomodulatory effects of UC-MSC-secreted factors on B-cells isolated from systemic lupus erythematosus (SLE) patients. The study provides novel insights into UC-MSC secretome-induced changes in molecules and receptors critically involved in B-cell activation, such as the B-cell activating factor (BAFF) receptor. The cell secretome also influenced B-cell function, antigen presentation, and survival. These findings could guide the development of future therapies for autoimmune diseases, including SLE, and other conditions involving dysregulated B-cell activity.

Baranwal et al. (Contribution 6) focused their review paper on advances in MSC-based treatments for enhancing arteriovenous fistula (AVF) patency. Vascular access and the patency of AVFs or AV grafts (AVGs) are essential for hemodialysis in patients with chronic kidney disease (CKD)/end-stage renal disease (ESRD), a condition that affects more than 10% of the global population. The authors summarized the involvement and capacity of MSCs or MSC-based products (e.g., exosomes, conditioned media) to regulate vascular remodeling, including immunomodulation, cell-to-cell contact, and paracrine factors. These MSC effects could be harnessed in future clinical studies to prevent AVF failure in CKD/ESRD patients relying on frequent hemodialysis. 

In summary, this Special Issue addresses the importance of MSC–immune cell–environment interactions and the significance of this crosstalk in the optimization of MSC therapeutic effects. Exploiting genetic and epigenetic alterations of MSC memory formation could change MSC fate and enhance their regenerative potential. Modifications of the MSC microenvironment could substantially delay aging, enhance tissue renewal, and allow for the use of MSCs or their exosomes in cancer treatment. Furthermore, activation of MAVS in MSCs and subsequent mitochondrial transfer to recipient cells could be used in MSC-based interventions against viral infections. MSC secretome-induced changes in B-cell activation could guide therapeutic advancements for disorders with dysregulated B-cell activity, including autoimmune diseases.

Therefore, exploiting and modifying MSC–immune cell–environment interactions could be used to advance cell therapies and to support the development of novel therapeutic approaches, personalized treatments, and efficient MSC use, especially in disorders and conditions that lack an effective causal treatment.
